# Risk of HBV Reactivation in Patients With Resolved HBV Infection Receiving Anti-CD19 Chimeric Antigen Receptor T Cell Therapy Without Antiviral Prophylaxis

**DOI:** 10.3389/fimmu.2021.638678

**Published:** 2021-07-15

**Authors:** Ping Li, Lili Zhou, Shiguang Ye, Wenjun Zhang, Junbang Wang, Xiaochen Tang, Jie Liu, Yangyang Xu, Wenbin Qian, Aibin Liang

**Affiliations:** ^1^ Department of Hematology, Tongji Hospital of Tongji University, Shanghai, China; ^2^ Department of Hematology, The Second Affiliated Hospital, College of Medicine, Zhejiang University, Hangzhou, Zhejiang, China

**Keywords:** CAR-T cells, B-cell malignancies, resolved HBV infection, HBV reactivation, immunotherapy

## Abstract

**Background:**

Chimeric antigen receptor (CAR) T-cell therapy has emerged as a novel treatment modality** for hematologic malignancies and is predicted to experience widespread use in the near future. However, not all risks associated with this novel approach are well defined. There are few data in the risk of HBV reactivation and limited experience in management in patients with resolved HBV infection who undergo CAR-T cell therapy.

**Methods:**

We performed a post-hoc analysis of a prospective clinical trial of anti-CD19 CAR-T (CART19) cell therapy in patients with relapsed or refractory (r/r) B-cell malignancies, and aimed at exploring the actual risk of HBV reactivation in a cohort of patients with resolved HBV infection receiving CART19 cell therapy in the absence of antiviral prophylaxis.

**Results:**

In this study, we investigated the risk of HBV reactivation after CART19 cell therapy in 30 consecutive patients with B-cell malignancies and resolved HBV infection without antiviral prophylaxis, in the Tongji Hospital of Tongji University. In this cohort, two patients developed HBV reactivation 2 months and 14 months after CAR-T cell infusion, respectively, the latter of whom developed severe hepatitis. These findings showed that the incidence of HBV reactivation was 6.67% (95% CI, 0.8–22.1). Specifically, none of the 21 patients who were HBsAb positive (0.0%) *versus* two of nine patients who were HBsAb negative (22.2%) experienced HBV reactivation (p = 0.03), suggesting HbsAb seronegativity at baseline is a possible risk factor in this population. Although use of tocilizumab or corticosteroids has been associated with increased risk of HBV reactivation, none of the patients who received these agents had HBV reactivation in this study.

**Conclusion:**

This is the first and largest study to assess the true incidence of HBV reactivation in patients with resolved HBV infection receiving CART19 cell therapy without antiviral prophylaxis. This study highlights that this population are at risk of developing HBV reactivation and indicates that close monitoring of HBV DNA is required in the absence of antiviral prophylaxis. In addition, antiviral prophylaxis is recommended in the HBsAb-negative subpopulation.

## Introduction

Hepatitis B virus (HBV) reactivation following chemotherapy or immunosuppressive therapy is a well-known and potentially fatal complication in patients with hematological malignancies, not only in HBsAg-positive patients, but also in patients with resolved HBV infection who are defined as seronegative for HBsAg but seropositive for HBcAb and/or HBsAb (1–3). HBV reactivation is best characterized in patients who are treated with B cell-depleting agents, such as rituximab. The rate of HBV reactivation during rituximab-containing therapy ranges from 16 to 80% in HBsAg+ patients and from 3 to 41.5% in HBsAg−/HBcAb+ patients without antiviral prophylaxis (2–5).

Adoptive cellular immunotherapy with chimeric antigen receptor (CAR) T cells has emerged as a novel treatment modality for hematologic malignancies (6–8). Three CD19-directed CAR-T (CART19) cell products, tisagenlecleucel, axicabtagene ciloleucel, and brexucabtagene autoleucel have been approved by the U.S. FDA for the treatment of children with acute lymphoblastic leukemia (ALL) and adults with advanced large B-cell lymphoma (LBL) and mantle cell lymphoma (MCL), respectively. With more approvals of CAR-T cell products and clinical indications evolving in the field of B-cell malignancies, CAR-T cells are anticipated to be used in an increasing number of patients. However, CAR-T cell-related toxicities are not fully elucidated and remain an important concern.

Given the similar mechanism of direct action on B-cells as rituximab, CAR-T cells may predispose HBV immune patients to HBV reactivation. China is a hyperendemic country for HBV infection, and many B-cell malignancies have a significant association with HBV infection (9). Not surprisingly, a high incidence and a fatal case of HBV reactivation in Chinese HBsAg carriers has been reported recently, which stimulates full vigilance about this potential risk (10, 11). However, currently, the risk and severity of HBV reactivation in patients with resolved HBV infection who undergo CART19 cell therapy is unknown.

In this study, we performed a post-hoc analysis of a prospective clinical trial of CART19 cell therapy in patients with relapsed or refractory (r/r) B-cell malignancies, and aimed at exploring the actual risk of HBV reactivation in a cohort of patients with resolved HBV infection receiving CART19 cell therapy in the absence of antiviral prophylaxis.

## Patients And Methods

### Study Population

Consecutive patients treated with autologous CART19 cells in a phase I/II clinical trial (NCT02537977) at the Tongji Hospital of Tongji University between January 1, 2017 and December 31, 2019 who fulfilled the following criteria were included: patients ware seronegative for HBsAg but seropositive for HBcAb at baseline; patients had undetectable serum HBV DNA (<100 IU/ml) and normal liver enzyme at baseline; patients had not received prophylactic antiviral therapy; patients had been followed-up for more than 1 month after CART19 cell infusion.

### CART19 Cell Therapy

The CART19 cells in this study were manufactured according to the Good Manufacturing Practice in the Stem Cell Translational Research Center, Tongji Hospital of Tongji University. The structure of the CAR contains a murine anti-CD19 single-chain variable fragment, a 4-1BB costimulatory domain and a CD3ζ T-cell activation domain. Eligible patients for this therapy had relapsed or were refractory to their previous treatments, including autologous or allogenic hematopoietic stem cell transplantation (HSCT). They were diagnosed according to the World Health Organization classification for tumors of the hematopoietic and lymphoid tissues. Expression of CD19 on malignant B cells was confirmed by flow cytometry or immunohistochemistry. An Eastern Cooperative Oncology Group (ECOG) Performance Score of ≤2, normal organ function, measurable disease, and a life expectancy of 12 weeks or more were necessary for eligibility, whereas patients with uncontrollable infection, active graft-*versus*-host disease, or clinically evident neurological lesions were excluded. All patients except one older than 75 years underwent lymphodepleting chemotherapy with FC regimen (25 mg/m^2^ fludarabine and 300 mg/m^2^ cyclophosphamide daily for 3 days) on days -5, -4 and -3, followed by intravenous infusion of CART19 cells. Dosing of CAR-T cells was 1–3 × 10^6^ CAR-positive T cells per kilogram of body weight. Study protocols were approved by the Ethics Committee of Tongji Hospital of Tongji University and conducted in accordance with the principles of the Declaration of Helsinki. All enrolled patients provided written informed consent for the treatment and follow-up.

### Monitoring of HBV Reactivation

HBV serologic test including HBsAg, HBsAb, HBeAg, HBeAb and HBcAb, HBV DNA levels and liver function (ALT, AST, and TB levels) were screened at the time of evaluation for CAR-T cell therapy, and routinely performed at 1, 2, 3, 6 months after CART19 infusion, and every 6 months thereafter until additional therapy for disease progression or HSCT or death. Serum HBV DNA was measured by real-time viral polymerase chain reaction (PCR) in our center using an ABI 7300 real-time thermo-cycler (ABI 7300; Applied Biosystems, Foster City, CA, USA) with a lower limit of 100 IU/ml. HBV reactivation was defined as elevation of HBV DNA ≥100 IU/ml for two consecutive measurements. Hepatitis flare was defined as serum ALT level >3×upper limit of normal (ULN) or an ALT increase >100 U/L, and severe hepatitis was defined as an ALT increase >10 × ULN or TB >1.5 × ULN.

### Analysis

The baseline characteristics of patients collected included HBsAb, HBV DNA, age, gender, histological subtype and treatment modality (the use of anti-CD20 antibody, HSCT, etcetera). Response of subjects with ALL and non-Hodgkin lymphoma (NHL) after CAR-T cell therapy was evaluated using the National Comprehensive Cancer Network (NCCN) guidelines, version 1.2016. Progression-free survival (PFS) and overall survival (OS) were estimated as the time from first infusion to first relapse or death, respectively. The probabilities of OS and PFS were estimated by means of the Kaplan–Meier method and compared using the log-rank test.

Cytokine release syndrome (CRS) and neurologic toxicity (NT) were evaluated in accordance with criteria from the American Society for Transplantation and Cellular Therapy consensus (12). CRS and NT were considered to be severe if it was of grade 3 or higher. Other adverse events were graded according to the National Cancer Institute Common Terminology Criteria for Adverse Events (NCI CTCAE V.5.0). Statistical analysis was performed using Stata version 15 SE. *P* value <0.05 was considered to be statistically significant. The cut-off date for data collection was May 31, 2020.

## Results

### Patient Characteristics and Outcomes of CAR-T Cell Therapy

A total of 30 consecutive patients with resolved HBV infection receiving CART19 cell therapy were included in this study. Baseline characteristics of this cohort and outcomes of CART19 cell therapy are described in **Table 1** and **Supplementary Table 1**. The median age was 59 years (range: 14–81). 17 (57%) patients were male. There were nine (30%) patients with acute lymphoblastic leukemia (ALL), 13 (43%) with diffuse large B cell lymphoma (DLBCL), five (17%) with mantle cell lymphoma (MCL), and three (10%) with other lymphoma subtypes. Among them, nine (30%) patients were seronegative for HBsAb. No patients had any other liver disease at baseline. During their prior therapy, all lymphoma patients received rituximab and four patients underwent HSCT.

**Table 1:** Baseline characteristics and responses to CAR T-cell therapy in the included patients.

Characteristic at baseline (n = 30)	
Median age, years (range)	59 (14–81)
Male	17 (57)
Hematologic diagnosis	
ALL	9 (30)
DLBCL	13 (43)
MCL	5 (17)
Others^#^	3 (10)
Previous HSCT*	4 (13)
Previous Use of anti-CD20 antibody	21 (70)
HBsAb-negative	9 (30)
Outcomes	
Objective remission rates	24 (80)
Grades 3–4 CRS	3 (10)
Grades 3–4 NT	0 (0)
Tocilizumab use	8 (27)
Corticosteroid use	6 (20)

Data are presented as n (%) of patients unless indicated otherwise.

ALL, acute lymphoblastic leukemia; DLBCL, diffuse large B-cell lymphoma; MCL, mantle cell lymphoma; HSCT, hematopoietic stem cell transplantation; CRS, cytokine release syndrome; NT, neurologic toxicity.

^#^Includes two high-grade lymphoma, and one chronic lymphocytic leukemia with Richter’s transformation.

*Three patients and one patient underwent auto-HSCT and allo-HSCT, respectively.

**Figure 1:**
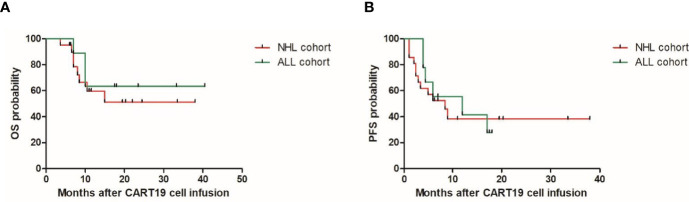
Overall survival (OS) **(A)** and progression free survival (PFS) **(B)** in the patients with resolved HBV infection after CAR-T cell therapy. ALL, acute lymphoblastic leukemia; NHL, non-Hodgkinlymphoma.

Previously, our group had reported the use of CART19 cells in patients with r/r B-NHL demonstrating activity (13, 14). In this population with HBV resolved infection, comparable response and survival rates were achieved (**Figure 1**). The overall response rate was 80%, with 70% of patients achieving complete remission (CR) (nine of nine with ALL, and 12 of 21 with NHL) and 10% (three of 21 with NHL) achieving partial remission. Among the 21 patients with NHL, the median OS was not reached and the median PFS was 8.5 months (95% confidence interval (CI), 3.8–13.2) with a median follow-up of 19.5 months (range: 3.5–38); the estimated probabilities of OS and PFS at 12-months was 59.7% (95% CI, 33.1–78.6) and 38.2% (95% CI, 16.5–59.8), respectively. Among the nine patients with ALL, the median OS was not reached and the median PFS was 12 months (95% CI, 1–26.6) with a median follow-up of 23.5 months (range: 7–40.5); the estimated probabilities of OS and PFS at 12-months was 63.5% (95% CI, 23.6–86.6) and 41.7% (95% CI, 10.9–70.8), respectively. There were three (10%) ALL patients who experienced severe CRS and no patients developed severe NT. Eight (27%) patients were treated with tocilizumab and 6 (20%) patients with corticosteroids for CRS or NT.

### HBV Reactivation and Hepatitis

**Figure 2:**
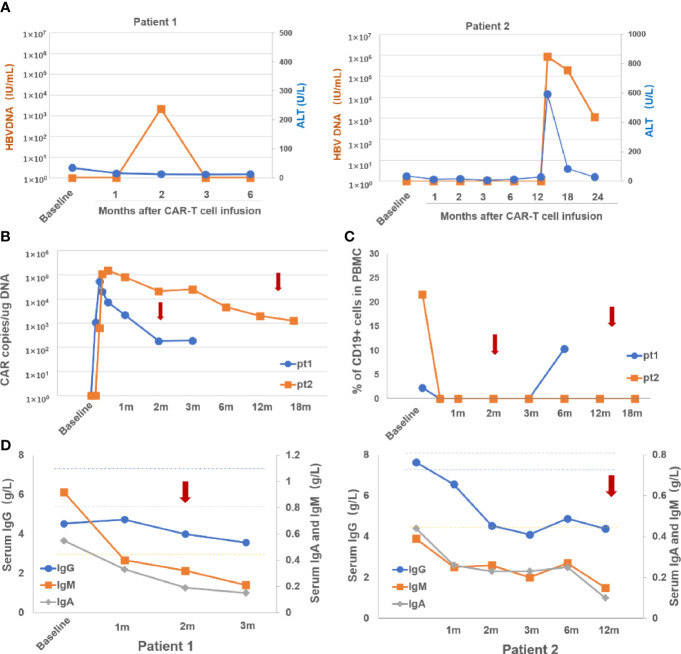
Dynamic changes o fHBVDNA and ALT **(A)**, CARcopies/ug DNA **(B)**, percentage of CD19+ Bcells in PBMC **(C)** and serum levels of IgG, IgA and IgM **(D)** after CAR-Tcell infusion in the two patients with HBV reactivation. Arrows indicate the time at HBV reactivation in the two patients. m, month; pt, patient; PBMC, peripheral blood mononuclear cells; Ig, immunoglobulin.

In this study cohort, two patients developed HBV reactivation 2 months and 14 months after CAR-T cell infusion, respectively, the latter of whom developed severe hepatitis. Neither of the two patients had received HBV vaccination and a history of active HBV infection. Both of them had antiviral prophylaxis during prior rituximab-containing chemotherapy. Details of the two patients with HBV reactivation are described in **Table 2** and **Figure 2**. With a median follow-up of 12 months (range: 2–37), the rate of HBV reactivation in this cohort was 6.7% (95% CI, 0.8–22.1). Specifically, none of 21 patients (0.0%) who were HBsAb positive experienced HBV reactivation *versus* two of nine patients (22.2%) who were HBsAb negative (p = 0.03). Although use of tocilizumab or corticosteroids has been associated with increased risk of HBV reactivation (15, 16), none of the patients who received these agents had HBV reactivation.

**Table 2:** Details of the 2 patients with HBV reactivation.

Patient no.	Age (years)/ Sex	Disease type/Ann Arbor stage at diagnosis	Refractory disease	Previous ASCT	Time from last use of rituximab to CAR-T cell therapy (months)	HBV status and hepatitis at baseline			Toxicity and arrangement after CAR-T cell therapy				Outcomes		
						HBcAb	HBsAb	ALT (U/L)/TB (umol/L)	CRS	NT	B cell aplasia (months)	use of tocilizumab and (or) corticosteroid	Response to CAR-T cells	PFS (months)	OS (months)
1	50/F	DLBC/IE	No	Yes	18	Positive	Negative	12/6.7	0	0	6	No	CR	9	15
2	59/M	DLBCL/IV	Yes	No	60	Positive	Negative	35/10	1	0	24	No	CR	24^+^	24^+^

F, female; M, male; DLBCL, diffuse large B-cell lymphoma; ASCT, autologous stem cell transplant; HBsAg, hepatitis B surface antigen; HBcAb, hepatitis B core antibody; ALT, alanine transferase; TB, total bilirubin; CR, complete remission; CRS, cytokine release syndrome; NT, neurologic toxicity; PFS, progression-free survival; OS, overall survival.

^+^Indicates ongoing response status.

The first patient was a 50-year-old female who was diagnosed with DLBCL 3 years before enrollment and relapsed after two lines of therapy and auto-HSCT. At baseline, the HBV serology showed HBcAb-positive and HBsAb-negative. She achieved CR by 3 months, which lasted 9 months after CAR-T cell infusion. She did not experience CRS or NT. Quantitative PCR showed that anti-CD19 CAR transgene was detected in peripheral blood mononuclear cells from 4 days to 12 weeks after CAR-T cell infusion. Blood CD19+ B cells became undetectable after lymphodepleting chemotherapy and remained absent until 6 months after CAR-T cell infusion. The levels of serum immunoglobulins (Ig’s), including IgG, IgA, and IgM were below normal levels at baseline, and decreased even further after CAR-T cell infusion. At the 2-month follow-up evaluation, she was found to have HBV reactivation (HBV DNA 2.18 × 10^3^ IU/ml) without ALT/TB elevation or HBsAg seroconversion, and was successfully treated with entecavir.

The second patient was a 59-year-old male who was diagnosed with DLBCL 6 years before enrollment and had relapsed after three lines of chemotherapy and radiotherapy. The patient was also HBcAb+/HBsAb− at baseline. Grade 1 CRS occurred after CAR-T cell therapy and did not require interventions with tocilizumab or corticosteroids. He achieved CR by 3 months after CAR-T cell infusion, which is ongoing after 24 months of follow-up. qPCR detected anti-CD19 CAR transgene from 7 days after CAR-T cell infusion, which was sustained in the blood for more than 1 years. Consistently, blood CD19+ B cells were absent from 7 days after CAR-T cell infusion. The levels of serum IgG, IgA, and IgM, decreased sharply by 41, 46 and 48%, respectively, during the first 3 months after CAR-T cell infusions, and have stayed at these low levels ever since. At 2 months after the patient finished the 12-month follow-up, that is, 14 months after CAR-T cell infusion, he was admitted to hospital with fatigue, vomiting and abdominal distension. He was found to have developed HBV reactivation (HBV DNA 8.53 × 10^5^ IU/ml with HBsAg seroconversion and severe hepatitis with a significant elevation of serum ALT level of 592.1 U/L (ULN 40 U/L) and TB level of 35.4 umol/L (ULN 21 umol/L). He was treated with entecavir which resulted in a gradual reduction of HBV DNA and ALT/TB. At the last evaluation for the 24-month follow-up, HBV DNA level was 1.11 × 10^3^ IU/ml, and ALT/TB levels were normal. He remains on entecavir afterwards.

## Discussion

There are few reports of HBV reactivation in patients with resolved HBV infection receiving CAR-T cell therapy in the literature. Strati P. reported a case of HBV reactivation after discontinuing antiviral prophylaxis in a DLBCL patient with HBsAg−/HBcAb+ at baseline receiving axicabtagene ciloleucel (17). Han et al. reported a case of HBV reactivation in eight patients with multiple myeloma and resolved HBV infection receiving CAR-T cells targeting BCMA (18). In contrast, three case series reported no reactivation among HBsAg−/HBcAb+ patients treated with single CART19 cell therapy (19–21). In two of the series, the proportion of patients with positive HBsAb was particularly high (90.9 and 85%, respectively) and some of them received prophylactic nucleotide therapy. Thus, the actual risk and severity of HBV reactivation in patients with resolved HBV infection receiving CAR-T cells remain unclear.

In this study, we investigated the occurrence of HBV reactivation after CART19 cell therapy in 30 consecutive patients with resolved HBV infection without antiviral prophylaxis. With a median follow-up of 12 months, the rate of HBV reactivation in this cohort was 6.67%. Given that the interval of HBV monitoring was not intense and our median follow up was relatively short in this study, the true reactivation rate in this population might be higher.

Although the use of entecavir in the two patients was effective in controlling HBV reactivation in this study, high HBV DNA level and severe hepatitis were observed at HBV reactivation in patient 2. In this study, HBV DNA was monitored every month in the first 3 months after CAR-T cell infusion and ≥3 months thereafter. This follow-up schedule may have contributed to the late detection of HBV reactivation and delayed instigation of antiviral treatment in these patients. Thus, monthly monitoring of HBV DNA could be important to identify patients in the initial stages of HBV reactivation and prevent HBV reactivation-related hepatitis and associated morbidity.

The time to HBV reactivation often occurs up to 12 months after the last dose of anti-CD20-containing therapy, however, delayed HBV reactivation (>12 months) still remains a concern (1, 22). Regarding CAR-T therapy specifically, so far, most of the published cases of HBV reactivation occurred within 6 months post CAR-T cell therapy. In this study, we observed a case of late reactivation occurring more than 1 year after CAR-T cell therapy. The rationale is that CAR-T cells as “a living drug” can persist in the blood for a prolonged period, which may cause long-lasting B-cell aplasia and a corresponding persistent reduction in immunoglobulin production (**Supplementary Figure 1**), thus prompting this late reactivation event (23). At HBV reactivation, the two patients had persistent blood CAR-T cells, absence of blood CD19+ B cells and hypoimmunoglobulinemia (**Figures 2B** **–D**), which further support the viewpoint. These observations also imply that monitoring the persistence status of CAR-T cells in the blood in addition to blood CD19+ B cells and serum immunoglobulin levels may assist to determine the optimum interval and duration of HBV DNA monitoring.

HBsAb seronegativity and prior treatment of rituximab have been identified as a risk factor for HBV reactivation in patients with HBV resolved infection (2, 24). In this study, as only two patients experienced HBV reactivation, it was not possible to determine clinical factors that could predict for HBV reactivation. Nevertheless, it is interesting to note that both patients were seronegative for HBsAb at baseline, which suggests this factor may also be associated with an increased risk of HBV reactivation in this population undergoing CART19 cell therapy. In view of the higher incidence of reactivation in HBsAb− patients compared to HBsAb+ patients in this cohort, prophylactic antiviral therapy may be advisable in such patients. It has been known that rituximab could induce B-cell depletion and also impact T-cell functions, which may facilitate HBV replication (25). However, the interval between the last use of rituximab and onset of HBV reactivation in the two patients was longer than 12 months, suggesting that rituximab is a less likely sole contributor to reactivation in our study. Yang et al.’s study further showed that the duration of prior rituximab treatment or the interval between rituximab-based chemotherapy and CAR-T cell therapy in a group of patients has not been associated with the risk of HBV reactivation (10).

## Conclusions

This is the first and largest study to assess the true incidence of HBV reactivation in patients with resolved HBV infection receiving CART19 cell therapy without antiviral prophylaxis. This study highlights that this population are at risk of developing HBV reactivation and indicates that close monitoring of HBV DNA is required in the absence of antiviral prophylaxis and monitoring the persistence status of blood CAR-T cells may help to determine the monitoring duration of HBV DNA. In addition, antiviral prophylaxis is recommended in the HBsAb-negative subpopulation. In order to design individual strategies to prevent HBV-related hepatitis following CART19 cell therapy, further large-scale studies are needed to identify risk factors and define the optimal duration and intervals of HBV monitoring in this population.

## Data Availability Statement

The raw data supporting the conclusions of this article will be made available by the authors, without undue reservation.

## Ethics Statement

The studies involving human participants were reviewed and approved by Wu Wenyuan and Le Junren. The patients/participants provided their written informed consent to participate in this study. Written informed consent was obtained from the individual(s) for the publication of any potentially identifiable images or data included in this article.

## Author Contributions

PL, LZ, SY, WQ, and AL were responsible for study design, data interpretation, and data analysis. PL wrote the manuscript. All authors recruited patients and contributed to data collection. All authors contributed to the article and approved the submitted version.

## Funding

This work was supported by funds from the National Natural Science Foundation of China (Nos. 81830004, 81830006 and 82070168) and Science Technology Department of Zhejiang Province (No. 2018C03016-1).

## Conflict of Interest

The authors declare that the research was conducted in the absence of any commercial or financial relationships that could be construed as a potential conflict of interest.

## Supplementary Material

Supplementary Figure 1 Dynamic changes of CAR copies/ug DNA, percentage of CD19+ B cells in PBMC and serum levels of IgG, IgA and IgM after CAR-T cell infusion in the patients without HBV reactivation. D, day; m, month; PBMC, peripheral blood mononuclear cells; Ig, immunoglobulin.

## Abbreviations

HBV, hepatitis B virus; HBsAg, hepatitis B surface antigen; HBsAb, hepatitis B surface antibody; HBeAg, hepatitis B e antigen; HBeAb, hepatitis B e antibody; HBcAb, hepatitis B core antibody; ALT, alanine aminotransferase; AST, aspartate aminotransferase; TB, total bilirubin.
