# Supplementary figures and images for: Risk of HBV Reactivation in Patients With Resolved HBV Infection Receiving Anti-CD19 Chimeric Antigen Receptor T Cell Therapy Without Antiviral Prophylaxis
Source: Front Immunol. 2021 Jul 15;12:638678. doi: 10.3389/fimmu.2021.638678 (PMC8320511; doi:10.3389/fimmu.2021.638678)

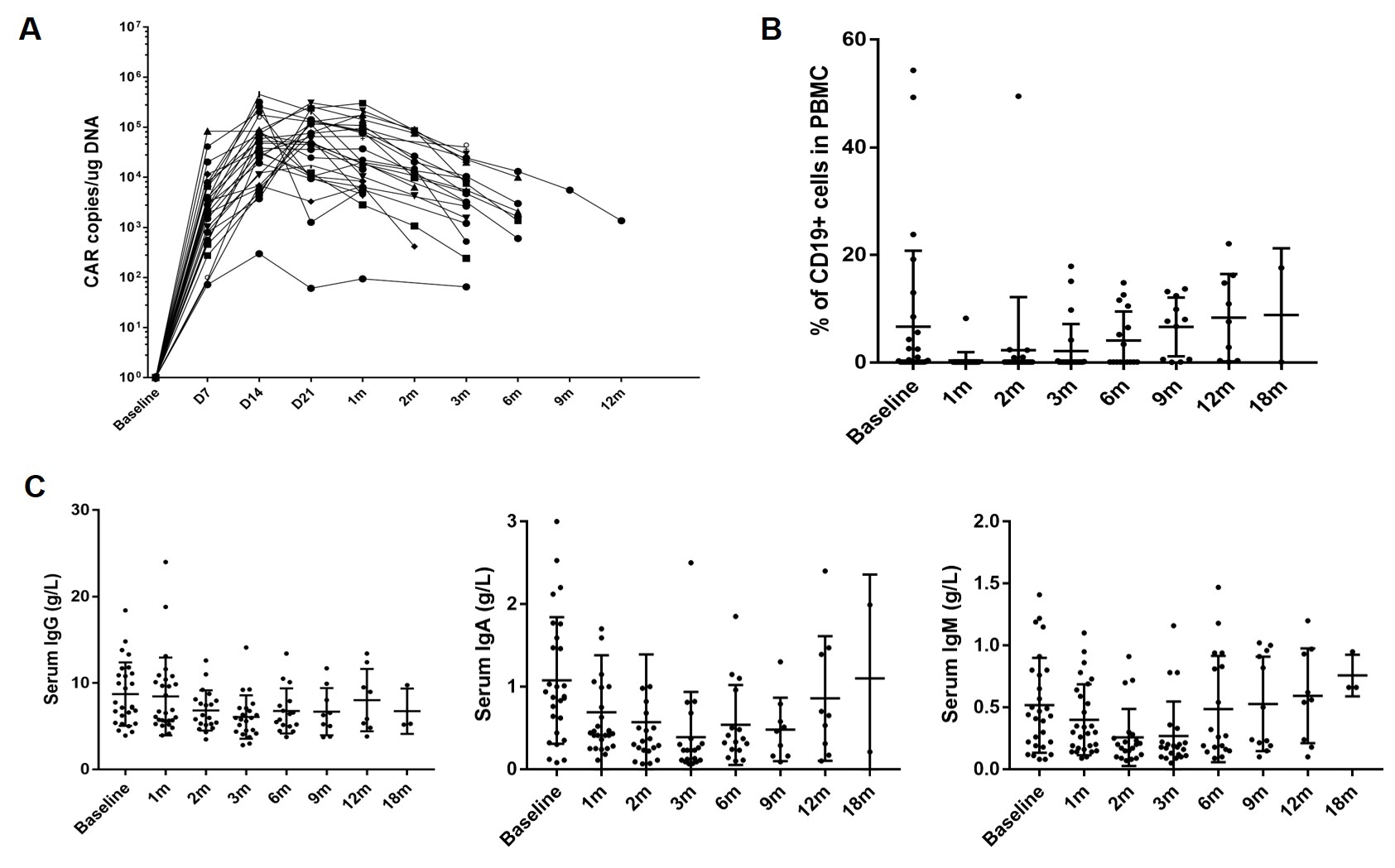

Supplement: Supplementary Figure 1 — Dynamic changes of CAR copies/ug DNA, percentage of CD19+ B cells in PBMC and serum levels of IgG, IgA and IgM after CAR-T cell infusion in the patients without HBV reactivation. D, day; m, month; PBMC, peripheral blood mononuclear cells; Ig, immunoglobulin. [file Image_1.jpeg]
