# Supplementary material for: Risk of HBV Reactivation in Patients With Resolved HBV Infection Receiving Anti-CD19 Chimeric Antigen Receptor T Cell Therapy Without Antiviral Prophylaxis
Source: Front Immunol. 2021 Jul 15;12:638678. doi: 10.3389/fimmu.2021.638678 (PMC8320511; doi:10.3389/fimmu.2021.638678)
Supplement: Supplementary file 2 [file Table_1.docx]

**Supplementary Table 1. Baseline demographics, disease characteristics, HBV serological status, conditioning regimen, CAR-T cell dose, response and toxicity after CAR-T cell therapy, and duration of follow-up for HBV reactivation in each patient**

Pt, patient; F, female; M, male; ECOG, Eastern Cooperative Oncology Group; DLBCL, diffuse large B-cell lymphoma; MCL, mantle cell lymphoma; HD NHL, high-grade non-Hodgkin lymphoma; ALL, acute lymphoblastic leukemia; HSCT, hematopoietic stem cell transplantation; Y, yes; N, no; FC: fludarabine/cyclophosphamide; C: cyclophosphamide; CR: complete remission; PR: partial remission; SD: stable disease; NR: no response. CRS, cytokine release syndrome; ICANS: immune effector cell-associated neurotoxicity syndrome.

*, indicates the patients with HBV reactivation post CAR-T cell therapy
